# Supplementary material for: Risk Factors, Prevalence, and Outcomes of Invasive Fungal Disease Post Hematopoietic Cell Transplantation and Cellular Therapies: A Retrospective Monocenter Real-Life Analysis
Source: Cancers (Basel). 2023 Jul 7;15(13):3529. doi: 10.3390/cancers15133529 (PMC10341183; doi:10.3390/cancers15133529)
Supplement: Supplementary file 1 [file cancers-15-03529-s001.zip › cancers-2431511-supplementary.pdf]

Table S1. Possible IFD in autologous HCT recipients.

IFD	Underlying Disease	Localization of Infection	Causative Agent	Outcome
Patient 1	HD	Lungs	Aspergillosis	Resolved
Patient 2	NHL	Central catheter	Candidemia	Resolved
Patient 3	NHL	Lungs	Aspergillosis	Resolved

HCT, hematopoietic cell transplant; IFD, Invasive fungal disease; HD, Hodgkin's disease; NHL, non-Hodgkin's lymphoma.
